# Should I Stay or Should I Go? International Students’ Decision-Making About Staying in Canada

**DOI:** 10.1007/s12134-021-00825-1

**Published:** 2021-04-01

**Authors:** Elena Netierman, Lauren Harrison, Angela Freeman, Grace Shoyele, Victoria Esses, Christine Covell

**Affiliations:** 1 School of Public Health and Health Systems, University of Waterloo, LHN 3721, 200 University Ave W. Waterloo, Ontario, N2L 3G1 Canada; 2 School of Nursing, University of Alberta, Alberta, Edmonton, Canada; 3 Department of Psychology, University of Western Ontario, London, Ontario Canada

**Keywords:** International students, Canada, Migration decision-making, Qualitative

## Abstract

Recent decades have seen an increase in the popularity of international education. Approximately 500,000 international students were in Canada in 2018 and this number is projected to grow. While we know that many international students decide to stay in Canada, we do not fully understand the decision-making process employed by international students regarding staying in Canada or going back home after completing their education. The purpose of this study was to explore how international students make decisions about their post-graduation destination and what factors they see as pivotal in shaping their decision-making process. We utilized a symbolic interactionist approach to analyze qualitative semi-structured interviews with 60 international students enrolled in post-secondary programs in Canada. Our findings suggest that the meaning students attach to staying in Canada varies from obtaining permanent residency to working for a few months upon graduation. We also demonstrate that for most students, the decision to stay in Canada is formed gradually and is shaped by familial obligations, cultural climate they experience in Canada, employment opportunities available to them upon graduation, and the possibility of obtaining permanent residency.

## Introduction

Over the last few decades, there has been a considerable increase in the popularity of international education worldwide (Beine et al., 2014). The number of international students more than doubled in the past 20 years, increasing from two million in 2000 to 5.3 million in 2017 (Migration Data Portal, 2020). About 75% of mobile students choose countries in the Organization for Economic Co-operation and Development (OECD) to pursue their education (OECD, 2014).

Canada has also experienced an increase in the number of international students in the tertiary education system since the 1990s (Lu & Hou, 2015) with international students representing 16.5% of the total number of students enrolled in Canadian schools in 2018 (ICEF, 2019). Currently, Canada is considered a top destination country for international education, along with the USA, the UK, China, and Australia (Canadian Bureau for International Education [CBIE] 2018a; ICEF, 2019; International Education Association of Australia, 2020). In 2018, 572,415 international students were enrolled in Canadian post-secondary institutions, and this number is projected to grow (CBIE, 2019). The economic contributions of international students to the Canadian economy reached $15 billion in 2017 in both direct and indirect costs (Global Affairs Canada, 2018).

Canada is attractive to international students due to its high-quality education system and its international reputation of being a safe country that promotes tolerance and takes pride in cultural diversity (Esses et al., 2018). Some researchers suggest that students’ choice of university and host country may also be impacted by the ease of obtaining a permanent residency status (Arthur & Flynn, 2013). Thus, it is possible that Canada’s immigration policies have become an added attraction to international students (Geddie, 2013). An ongoing commitment to promote international education was demonstrated by the Canadian government in the 2019 budget, which dedicated $147.9 million over 5 years to the “International Education Strategy” (Gov Can Budget 2019).

While international students are considered to be visitors to Canada, some of them may decide to stay in the country upon graduation. The results of the 2018 survey of the CBIE (2018b) showed that approximately 70% of international students planned to stay and work in Canada upon graduation and 60% intended to apply for permanent residency. International students have been described as “designer immigrants” due to their familiarity with Canadian culture and locally obtained training (Hawthorne, 2012). Although Canadian policy makers are cautious about actively recruiting international students to stay in Canada due to ethical concerns related to such practices (Neiterman et al., 2018), favorable immigration policies (Lu et al., 2009; She & Wotherspoon, 2013) may indirectly entice international students to stay in Canada upon graduation. Canadian immigration policies support the integration of international students into the labor market by allowing students to work on- and off-campus (Government of Canada, 2020a). Additionally, graduating students can apply for a Post-Graduation Work Permit, which, in some cases, is valid for up to 3 years (Government of Canada, 2020b). Work experience gained while studying enables interested students to apply for permanent residency utilizing the path for Canadian Experience Class (Government of Canada, 2020a). Canadian education may provide international students with a higher-ranking position in the Express Entry candidates’ pool (Government of Canada, 2020c) and speed up the process of receiving permanent residency.

Given the growing interest from many countries, including Canada, in providing education to international students and the possibility that some of these students may decide to stay in the receiving country upon graduation, it is important to understand how international students arrive at the decision to go back home or stay in their country of education. This knowledge can inform human resources and immigration policies as well as aid with developing supports for students planning to permanently stay in the country of education. While the factors influencing migration-related decisions of international students have been explored to some extent in the literature (Alberts & Hazen, 2005; Esses et al., 2018; Farivar et al., 2019; Geddie, 2013), most of them have been derived from quantitative studies. Consequently, they show associations between variables shaping students’ decisions, but do not shed light on the decision-making *process* (Szelényi, 2006; Wu & Wilkes, 2017). Moreover, most of the literature conceptualizes students’ migratory transitions as unidirectional (e.g., going home vs. staying in the host countries), which simplifies the potential complexity of migration pathways available to international students who may decide to move to another country upon graduation (Wu & Wilkes, 2017). We also know little about the *meaning* that students attach to the decisions related to migration and how this meaning-making process impacts their migration choices. The goal of this paper is to address these gaps by exploring how international students think about and arrive at a decision to stay in Canada or go back home after graduation.

## International Students’ Decision-Making

At present, there is no consensus in the literature about how and when students make decisions about immigration. Although Lu et al. (2009) found that students’ original intentions regarding permanent migration prior to relocating largely predicted their intention to stay, other work has shown that students’ feelings change and may alter their intentions (Alberts & Hazen, 2005; Basford & van Riemsdijk, 2017; Farivar et al., 2019). Most likely, students’ decisions are made on an ongoing basis whereby students assess and reassess their migration options (Hazen & Alberts, 2006). Midway through their programs, many students studying abroad find themselves considering their migration decisions post-graduation (Alberts & Hazen, 2005). Furthermore, the final migration decisions of students may differ from their previous intentions altogether (Baruch et al., 2007). While students may or may not change their mind, migration is a dynamic process which most likely cannot be reduced to a single definitive decision (Wu & Wilkes, 2017). It seems that the decision to immigrate is a complex transition that may change over time. This study aims to explore this process by offering insights about the meaning international students attach to migration pathways available to them upon graduation.

Migration studies often classify students’ options regarding migration as either short-term stays or long-term permanent moves (Sage et al., 2013). This can create challenges for studying the sequential movement of students as they are a highly mobile group that may not fall into these two categories (Sage et al., 2013). For example, it is possible that after graduation, students choose to move to a third country, neither staying in the country of education nor going back home (Szelényi, 2006; Wu & Wilkes, 2017). The assumption that students either permanently reside in their receiving country or leave it to return back home after graduation also neglects the fact that some international students may choose to stay in the receiving country for only a few years after finishing their degree. Students may want more time to make decisions, or they may stay to gain work experience and save money before returning home (Alberts & Hazen, 2005; Hazen & Alberts, 2006). Additionally, some students plan to stay in the receiving country for short-term work experience as a continuation of their graduate degree in the form of professional development (Szelényi, 2006). Whether or not a student decides to stay or leave upon graduation could entail a wide variety of migration plans and is likely linked to the meaning students attach to migration, their relationships with significant others in the receiving and home countries, and a variety of professional and personal circumstances (Geddie, 2013; Szelényi, 2006).

In order to understand the decision-making process of international students, some researchers utilize the push-pull factors model, which contextualizes migration-related decisions as an interplay of factors that deter people from home countries (push factors) and attract them to receiving countries (pull factors) (Mazzarol & Soutar, 2002). The push-pull model has been also applied to analyze migration of Canadian international students. For example, the retention of English-speaking students (both foreign-born and domestic) in Quebec was explored using a push-pull model, which enabled researchers to identify a variety of factors that shaped students’ decision-making, including good job opportunities as a factor that may push students to leave the province, and cost of living as a factor that may pull them to stay (Holley, 2017). Understanding the decision-making process and utilizing the push-pull model are complementary and have been used congruently to explore the choice of graduate school by East Asian students in Ontario (Chen, 2007). While the push-pull model has provided useful insights into social, political, and economic factors that shape students’ decisions, it has been criticized for providing an overly simplistic model that does not account for characteristics of the individual (Lee, 2017) or their social relationships (Geddie, 2013). This paper will build on this knowledge by exploring the micro-level process of decision-making.

Overall, a growing body of literature shows the complexity of migration intentions of students (Esses et al., 2018; Lu et al., 2009). Researchers quantified the outcomes of students’ decisions to migrate post-graduation (Lu & Hou, 2015) and identified and measured factors considered by students making their decisions (Baruch et al., 2007, Findlay 2011). Qualitative studies contributed to this body of knowledge by exploring the interplay of micro-level factors weighed by students making decisions about migration (Alberts & Hazen, 2005; Geddie, 2013; Szelényi, 2006). There appears to be some consensus that migration decisions of international students are made over time and may unfold as a process (Alberts & Hazen, 2005; Farivar et al., 2019), but very little is known about students’ *experiences* of going through this process and the *meaning* they assign to it.

To address this gap and explore the process of decision-making employed by international students, this study uses a symbolic interactionist approach. Symbolic interactionism posits that individuals are engaged in the meaning-making process through social interactions (Blumer, 1969) and that they continuously assess the situations to determine their own interpretations of their circumstances and to form their actions (Oliver, 2012). The meaning that individuals attach to any social process is of particular importance in symbolic interactionism, as perceptions about social phenomena are seen as products of everyday interactions (Polk, 2017). The focus on meaning-making processes and micro-level understanding of individuals’ actions make symbolic interactionism a suitable approach for understanding how international students in Canada approach decision-making about staying in the receiving country or going back home. Employing symbolic interactionist theoretical perspective, the goals of this paper are to explore (1) how do international students make decisions about staying or going back home or another country upon graduation and (2) what do they see as pivotal in shaping their decision-making processes?

## Methodological Approach

After receiving ethics clearance from [BLINDED FOR PEER REVIEW], participants were recruited from two universities located in two different Canadian provinces by placing advertisements about the study on university campuses and utilizing snowball sampling. In total, 60 participants took part in the study (30 students per university). We aimed for diversity in our sample, recruiting undergraduate (*n*=18) and graduate (*n*=42) students who self-identified as female (*n*=39), male (20) or non-binary (*n*=1) individuals. In order to explore how the field of study may shape migration-related decision-making process for international students, we purposively recruited 20 students per field (10 students per site) studying in the general fields of (1) social sciences and humanities (SSH), (2) health sciences (HS), and (3) STEM (science, technology, engineering, and math). Our participants came from 23 different countries, with the highest number of students coming from China (*n*=18, 30%), India (*n*=11, 18.3%), and Iran (*n*=5, <1%). All participants were in their last year of study. Table 1 provides a summary of a demographic profile of our participants.

**Table 1:** Demographics study sample (*N*=60)

Age	*M* = 26.73, SD = 5.78, range =32
Gender	*N* (%)
Female	39 (65)
Male	20 (33)
Binary	1 (0.017)
**Country of origin**	*N* (%)
China	18 (30)
India	11 (18.3)
Iran	5 (0.083)
Ghana	3 (0.05)
Pakistan	2 (0.03)
Russia	2 (0.03)
United States of America	2 (0.03)
Vietnam	2 (0.03)
Austria	1 (0.017)
Bangladesh	1 (0.017)
Brazil	1 (0.017)
Colombia	1 (0.017)
Egypt	1 (0.017)
Hong Kong	1 (0.017)
Ireland	1 (0.017)
Japan	1 (0.017)
Malaysia	1 (0.017)
Nigeria	1 (0.017)
Saudi Arabia	1 (0.017)
Sri Lanka	1 (0.017)
Thailand	1 (0.017)
United Kingdom (England)	1 (0.017)
Zimbabwe	1 (0.017)
Year entering Canada for study	*M* = 2014, SD = 2.04, range = 13
Field of study	*N* (%)
Social Sciences and Humanities	20 (33)
Sciences, Technology, Engineering and Math (STEM)	20 (33)
Health Sciences	20 (33)
Level of study	*N* (%)
Undergraduate	18 (30)
Masters	18 (30)
PhD	24 (40)

**Fig. 1:**
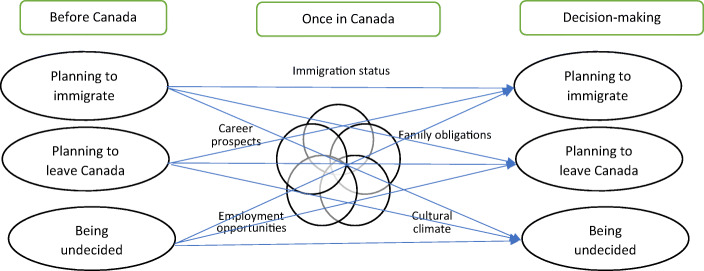
Process of decision-making of international students

Trained research assistants conducted semi-structured interviews with the students during 2017–2018 utilizing an interview guide which explored students’ decision-making prior to coming to Canada, their experiences in Canada, and their considerations about staying in Canada or returning back home upon their graduation. All interviews were conducted in English. As an incentive to participate in the study, students were offered a $30 gift card to a local grocery store. The interviews were recorded, transcribed verbatim, and analyzed using NVIVO 11 software for qualitative data analysis.

The qualitative data from this study were analyzed inductively, utilizing Charmaz’s (2014) guidelines. We felt that Charmaz’s (2014) focus on inductive, open-ended coding fits well with symbolic interactionist’s theoretical lens by enabling us to explore the process of students’ decision-making while also learning about their experiences and the meaning they attach to it. We started the process of data analysis with two researchers independently coding the data and moving from open, line-by-line coding to more focused coding. After coding five interviews, we developed a coding scheme that was subsequently applied by all the researchers to code the interviews and modified a number of times during the process. We then proceeded with the development of analytical categories, identifying the relationship between them and seeking to derive a more nuanced understanding of the processes employed by the international students in making decisions about staying in Canada or going back home. In what follows, we summarize the findings related to the way in which international students conceptualized the decision-making process, focusing on the complexity and uncertainty of this endeavor. We assigned pseudonyms to all the participants to protect their identities. Due to concerns with confidentiality, we do not always reveal some of the characteristics that can hint at the identity of the participants (e.g., level of study, province of study, and age). However, where possible, we provide some demographic details about the participants to help the readers place the quote in context.

## Results

### Broadening the Meaning of Staying

The vast majority of students who took part in our study had some plans in place following graduation to either stay in Canada or return back home, with most hoping to stay. But the meaning of “staying” varied considerably among the participants, ranging from short- to long-term planning. While over 50 students planned on “staying,” only 10 of them saw “staying” after graduation as permanently relocating to Canada. Conversely, over one-third of the participants planned to “stay” in Canada for a few years and then move back home. For example, Alix, a 23-year-old undergraduate student from China, said:

I plan to stay in Canada for a few years before I go back to China. I’m not sure if I will really go back to China after I stayed in Canada for, like, many years. But for now, my plan is [to] stay in Canada for about, like, three or four years, if possible... [I am] planning to stay in Canada and find jobs and gain some working experiences and then, probably, go back to China. And kind of, like, bring new skills –new knowledge, to my country. [Laughing] Something like that. (Alix, a 23-year old, STEM undergraduate student from China)

As Alix suggests, while she has a “plan” to stay in Canada, she is not set on how many years she will be here—her plan is flexible, with the possibility that it will change considerably as she continues to live and work in Canada. Her notion of “staying” is not linked to the permanent desire to remain in Canada. Rather, it is built on her intention to prolong her stay for a few additional years. Similarly, Chang, a 24-year-old, STEM graduate student, talked about staying for a period of time in Canada before going back to China as a general plan that does not have specific, well-defined timelines:

So,.. I think ah, yeah. [For] short … period of time, I will… like, find a job and … get more working experience here in Canada. And maybe, in two years I will return to China to … build my career back there. Because all my … family are in China. And many of my friends are also in China. So, I hope I can return there.

The uncertainty of Chang’s plan is evident in his hesitation to define what “staying” means to him. He refers to thinking about “maybe” staying in the receiving country for a “period of time,” but is also hoping that eventually he will go back home. Like Chang, many participants talked about their intention to stay in Canada for some time in order to gain work experience or pay off student-related debts prior to returning home, signaling a possible desire to return home in a position of improved financial security or increased employability.

For more than half of participants, staying in Canada included a plan to apply for Permanent Residency (PR) at some point following graduation. The students often identified a few benefits of obtaining PR status, including improved access to scholarships, education and increased job opportunities. Students also discussed how obtaining PR status would increase flexibility in decision-making and their ability to move in and out of Canada. Thus, applying for and getting PR status in Canada was seen as a way to keep the doors open, but did not necessarily indicate a firm decision to stay in the country. Betty, a 25-year-old from England working on her PhD research in a STEM field, noted:

I mean, I would… ideally like a form of residency that allows me to leave [Canada] and come back… ‘Cause, like, [work] in academia is an inherently transient career. … it would be nice to have the option to kinda go and do a post-doc, you know, say somewhere in Europe or something.

Betty’s account suggests that the meaning she attached to getting permanent residency cannot be equated with a decision to stay in Canada full time—her goal is not to continuously and permanently live in Canada, but to expand her options for future travel and perhaps come back to Canada later. Betty’s position was similar to the sentiments shared by most of our participants—“staying” in Canada was not defined as a unidirectional trajectory of permanently settling in the new country. For some students, the meaning of staying meant delaying the return home for a few months or a few years. For others, “staying” signified obtaining permanent residency and having a legal right to stay and work in Canada. In both cases, however, students wanted to keep the door open and their view on “staying” or “going” had been sketched only using general contours, reflective of the uncertainty about their future plans.

### Making Decisions About Staying

Our analysis revealed that there was considerable variation in how international students made decisions about staying in Canada. Four students had contractual agreements with their countries that necessitated their return. A handful of students remained adamant that they had always planned to go back home after completing their education. These students usually cited family reasons and financial obligations as key factors in going back home. Hung, a 22-year-old undergraduate student in the Health Sciences field from Vietnam, said:

I know, like, some of my friends, even though they wanna stay, they can’t really stay because… their family – their family don’t support [them staying], and they have a business in the country. And they want the kid to come back to take over the business.

According to Hung, students’ personal desires are not necessarily a key factor in their ability to stay in the country. Coming from cultures that emphasize filial piety, some students saw their familial obligations as pivotal for their decision to go back home, even when their own preference would be to stay in Canada. As such, the mobility of these students was not necessarily defined by the decision-making process, but by a set of obligations related to students’ family roles. For others, the autonomous decision-making was still a part of the process, even when it included familial pressures. “I’m really torn. I… want to stay. I do. But I also made a vow to my parents years ago that I would take care of them as they were aging,” noted Layla, a 30-year-old graduate student in Health Sciences field from the USA, reflecting on this personal struggle.

While some of our participants did not plan to stay in Canada upon completion of their degree, ten students had a strong desire to remain in Canada, which was formed long before their arrival. For this group, the decision to immigrate upon graduation played a key role in choosing Canada as a country of destination. Rania, a 22-year-old female student from Malaysia studying in Social Sciences and Humanities field, explained:

I think I’ve made up my mind even before I came here for my first year… Because I visited Canada back in 2009 when I was still in the middle of high school… I really like it here. So…that’s why – that’s also – big reason why I chose to study at [UNIVERSITY], is to… stay close to family and also stay here.

As Rania pointed out, her decision to stay in Canada was made prior to arriving in the country. Participants who said they had made a firm decision to stay in Canada even before they arrived often entwined the decision to study in Canada with an intention to emigrate from their home country. Marina, a 25-year-old Social Sciences and Humanities student from Russia, explained:

My original purpose was actually to immigrate to Canada and I was trying to find the best or the easiest way of doing it. And after doing some research, I realized that one of the best strategies for me would be to come and study here.

For Marina, studying in Canada was a means for obtaining permanent residency. Later in the interview, Marina also explained that, in Canada, the partner of an international student can get a work permit, whereas in other countries she explored this option was not available. As a result, Marina and her partner decided to choose Canada for their studies. Marina’s decision-making process about immigrating to Canada, therefore, was the one that prompted her to choose to study in Canada as an international student. Mei, a 30-year-old graduate student in the field of Social Sciences and Humanities who came from China, said “I know some of my classmates just come here for the PR. And the degree is just a way to get the PR,” suggesting that Marina’s immigration strategy is not unique. For students who arrived in Canada with a plan to stay in the country, the perceived complexity of the path from international studies to permanent residency was a key consideration in deciding to study in Canada, and the decision to immigrate was made prior to becoming a student.

Although nearly a quarter of the students made the decision about immigration prior to arriving in Canada, most students who took part in our study were not entirely certain what their plans for the future were before they had a chance to live in Canada. Exploring life in Canada while adapting to the local education and culture was seen as a strategy to make a more informed choice. Lin, a 28-year-old Social Sciences and Humanities PhD student from China, recalled:

I guess, in the first… two years, I wasn’t sure if I wanted to stay. Because – well, that was my first two years and I was still doing a lot of adjustments and everything. And, so I was thinking maybe other options are okay, as well. But then… after things got settled down, I just um – I just started to like this place and just wanna stay.

Lin’s description of her decision-making process is typical of the majority of students whom we interviewed. Most of our participants were not necessarily settled on a decision before arriving in Canada and felt that their experiences in Canada would provide a better platform to make an informed decision. Arriving at the final decision was tied to a number of factors that we describe in the following section.

### Confirming, Changing, or Delaying the Decision

Nearly two-thirds of our participants noted that while they had made an initial decision to stay/go back home before arriving in Canada, these decisions were either reaffirmed or changed after they began their studies. For instance, Moon, a 20-year-old female, Health Sciences student from Bangladesh, said that her decision to stay in Canada “solidified” after she arrived in Canada and “saw first-hand that this really is a great country. And it really is, you know, quite welcoming.” Twenty-six-year-old Arjun, an Engineering student from India, noted that his desire to stay in Canada “has actually increased over the period of time.” Wei, a 20-year-old female undergraduate student from China, explained:

Um, I will say over the time I… willing to stay, get stronger and stronger. Yeah. Hmm, I think the more I learn, [the more] I find … like, opportunity I have is in Canada.

But not all students had positive experiences. Some students found that Canada lost its initial allure. Reflecting on the change in his decision to stay in Canada, Diego, a 27-year-old male PhD student from Brazil, commented:

You know, … before you come, you have this romantic view… I will never leave… Before you start, you don’t know exactly how things are going to go, and you think it’s going to be… you know, smooth forever. You know, I wasn’t exactly aware that Canada wasn’t one of the biggest countries in my field, you know, to stay after graduation. So yes, in – in the beginning I thought, wow, Canada forever. Um, but then again, I’m maybe too pragmatic.

As Diego pointed out, with time, his view of Canada has changed from being “romantic”—feeling committed to stay in Canada “forever”—to a more “pragmatic” view, which considers opportunities for future employment in the high-tech industry. For Diego, therefore, the decision to stay in Canada was altered by his view of the employment opportunities in Canada and in the global high-tech industry, and opportunities available to him for professional growth.

For other students, the decision to leave Canada upon graduation was tied to their experiences of social interactions with others. Mary, a 27-year-old Health Sciences student from Ghana, said:

Okay, so… initially when I came, I was thinking of staying. Because I felt this place was more peaceful. And then, you have the opportunity to develop yourself. You understand? But then, as time went by, and then I became exposed to the realities here, I feel very discouraged from staying here.

In her interview, Mary talked about the difficulty of fitting in as a visible minority student. She recalled her challenges of getting used to what she termed a “Canadian” culture—a type of social interaction that Mary interpreted as distant and impersonal, “totally different” from the “community based” culture that was familiar to her. Mary felt that in Canada “people are in their individual space,” which, she admitted, came to her as a “shock.” Learning about Canada through social interactions with other students, Mary felt that she might not be welcomed in Canada. As with some other students who self-identified as members of visible minority groups, Mary recalled a number of instances where she experienced racism and discrimination. These encounters prompted her to re-evaluate her initial decision to stay in Canada and consider going back home.

Students identified a plethora of factors that shaped their decision to stay or go. These included ties to family members who were either in Canada or abroad, perceptions of employment opportunities in Canada and in one’s home country, and the perceived complexity of the immigration process. Some students also talked about feelings of social isolation and loneliness that they experienced in Canada. Chun, a 21-year-old female, STEM undergraduate student from China, recalled:

The first year, which is the – the year that I got homesick… It’s my first year. Leave my parents. And living alone… is hard for me. So, at that time, I was wish[ing]… after I finish my university here… go back to China. I’m gonna stay with them [my parents]... Well, after the first year, everything [is] getting better, ‘cause, like, I get into like local environment. I get used to speak up... [to] local people. And I got used to the lifestyle here. Ah, it’s getting easier for me to… enjoy the life here. So, after I get into university, I found that, “Wow, it’s a nice place. I want… I want to like, stay over longer.

Similar to some other participants, after spending the first few years in Canada, Chun found herself to be more immersed in the local life—she got used to the “local environment” and adapted to the Canadian culture by learning to “speak up” and to communicate with “local people.” Chun noted that being in university made her realize that she likes Canada and, as a result, Chun has been leaning towards staying in Canada for longer than she originally planned.

While some students attributed their decision to their feelings towards and attitudes about Canada, for others, the decision was linked to the economic and social conditions in Canada. “Quality of life is way better here. And quality of education – that was my first priority why I came over here,” noted Aisha, a 20-year-old undergraduate Health Sciences student from Pakistan. Banhi, a 26-year-old from India studying towards the completion of a Health Sciences graduate degree, identified another factor that swayed her decision in favor of staying in Canada:

Initially, I was, like, I’ll complete my degree. I’ll go back. But then, like, after my first year, perhaps halfway into my second year, I was, like, it’s good opportunity over here. Like, it’s different, but it’s good. So, I want to stay. That’s – that’s kind of when I started appreciating the system and the health care benefits I got.

Banhi described how her growing knowledge about Canada has shifted her decision about returning back home. This process is similar to the gradual change that was described by Jessica, a STEM PhD student from Zimbabwe:

First year I was there, I [said] … “I’m not staying.” And my friends were all laughing at me, the Zimbabweans, are, like, “Uh huh. But that’s how we all were when we came.” But after, you know, so many years, then they… want to stay. So, I think mine [decisions about staying] have changed, cause now I’m no more so… “I’m going back.” Now I’m like, “Okay”… I think there’s lots of possibilities… which is different from when I came over.

Although Jessica is not completely sure that she is planning to stay in Canada, her initial conviction that she wants to go back home after completing her PhD has somewhat faded. Similar to Banhi, her experiences in Canada compelled her to re-evaluate her original decision.

Finally, eight students felt that making decisions about staying in Canada or going back home can or should be postponed. Adaku, a 20-year-old, Social Sciences and Humanities undergraduate student from Nigeria, noted:

So, I think – but people definitely do change. Like, you often – like I think you keep it at the back of your mind, like what are your long-term plans? … I like to plan things out. I like having lists. So, I guess, like, that could be… me. And then there are people just like – at this point, I’m thinking of just, like, okay, you know what? Let’s just go with it and see what happens. Like, if you decide to, like, go, you go. If you decide to stay, you stay. But you can definitely change – like, you change your programs. You change – life happens. Your choices change. Um, your desires change and stuff, so…

Adaku discusses her thoughts about staying in Canada or going back home, but is somewhat reluctant to arrive at a specific decision. Reflecting on her experience, Adaku is questioning if her personality of liking to “plan things” and “having lists” can outweigh her appreciation of the fact that the future is very uncertain. Comparing a migration-related trajectory to the journey of studying, which often takes students onto unanticipated paths, Adaku suggested that the decision about migration may not be feasible during the time where students’ experiences are rapidly changing. For some, the refusal to commit to a decision was tied to the uncertainty about their legal status or family roles. For others, the lack of clarity about future employment was the reason for why the decision was not made. Reflecting on this state of uncertainty Mary, a 27-year-old Health Sciences Master’s student, said:

At this point… it’s very blurry. It depends on what [will] happen, okay? Because I feel …. when I’m done with my Master’s degree, getting that Canadian experience before I go [home] will be helpful, right? But then, if you don’t get any opportunity out there, then it’s better you go.

Mary’s feeling that her future plans are “blurry” is consistent with what some other participants described when they discussed their thoughts about migration. Although about half of our participants kept an open mind about the possibility of migration, students like Mary were uncertain about whether or not they will be relocating to Canada. Making decisions about their prospects of staying in Canada seemed pointless to these participants given the fact that they had no knowledge about their chances of getting permanent residency or their opportunities for gaining employment in Canada. In this context, the “decision-making process” involved not committing to any decision, leaving it for the time when the overall picture of the future would be more complete.

## Discussion

In this paper, we explored the meaning that international students attach to the decision-making process of staying in Canada or going back home upon graduation. We showed that the meaning of “staying” was defined by students in a multitude of ways—students saw “staying” not only as a permanent migration, but also as a temporary stay (for a few years) or a possibility of coming back to Canada as a permanent resident. Hence, “staying” and “going back home” were rarely seen by students as firm decisions with fixed definitions attached to them. Rather, students considered them as possibilities or opportunities which could represent short-term plans or long-term migration decisions.

Our findings also demonstrate that international students’ decision-making process regarding migration is complex and can change over time. Students’ migration-related decisions can be understood as falling into three broad categories. Figure 1 summarizes these categories and demonstrates how students’ initial decisions may be altered by the contextual factors (e.g., career prospects, employment opportunities, family obligation, and immigration status) that they encounter in Canada.

Consistent with the findings from the literature (Esses et al., 2018), we found that some students arrive in Canada with a firm decision to immigrate. In our study, only ten students had an initial strong desire to stay in Canada that remained unchanged while they studied. For these individuals, the very decision to get an international education was a means to immigrate to Canada. These students, therefore, could be regarded as potential immigrants who chose a unique path—education—for obtaining a permanent residency status.

The other, small group among our participants was comprised of those who planned to go back home upon completion of their education. Four of these students had contractual obligations with their governments. Other students within this group cited familial and personal obligations as the reasons forcing them to go back home. In this context, the “decision-making process” was not free and autonomous, as some students felt that their family needs are the ones that define their future place of residence (Geddie, 2013; Wu & Wilkes, 2017). For these students, international education was an opportunity to enhance their social or employment status in the home country. However, once coming to Canada, some of these students may have changed their views on the possibility of migration and some planned to prolong their stay after graduating to gain some employment experience before going back home.

Most of our participants, however, came to Canada with only a general idea about their future. Among the students we interviewed, approximately 2/3 thought about staying in Canada for at least a few years and over a half planned to apply for a permanent residency status. None of these participants had a firm stance on either staying in Canada or going back home and their experiences in Canada, such as difficulties with obtaining permanent residency status, lack of employment opportunities, or experiences of racism and discrimination, shaped their decision-making process (Dam et al., 2018).

Securing employment upon graduation was seen by international students who took part in this study as pivotal in shaping their decision to stay in the country (even if only temporarily). Given the interest of some countries to attract and retain international students to fulfill local human capital needs, it would be beneficial to establish clearly defined pathways that facilitate acquisition of work visas or permanent residency status for international students. Our study showed that some students choose to study in Canada because it offers options to either work or apply for permanent residency upon graduation (Government of Canada, 2020a). Other top destination countries for international education have similar policies. For instance, Australia allows international students to apply for Temporary Graduate visas which may simplify the pathway to permanent residency (Chew, 2019). Germany provides international student an option to secure a temporary residence permit which gives them 18 months to find a job in their field (Hoffmeyer-Zlotnik & Grote, 2019). However, as we indicated elsewhere [BLINDED FOR PEER REVIEW], information about immigration opportunities for international students is usually managed by hosting institutions, with some universities providing more access to information than others. Therefore, the process of obtaining work visas or permanent residence may be perceived by students as inaccessible and complex. This complexity was evident in our study through the high level of uncertainty expressed by international students about their ability to stay in Canada. Moreover, since migration in many countries, including Canada, is tied to the local economic needs, which position applicants with certain skills and qualifications as more desirable (Kapur & McHale, 2005) and is also shaped by cultural and political ideologies (Menz, 2009), some students may have easier access to immigration and post-graduation work than others. Evidently, while Canada has a global reputation for cultural tolerance (Esses et al., 2018), in our study the voices of students who self-identified as visible minorities highlighted the instances of racism and discrimination they experienced in Canada, which made some of them to seriously consider returning back home.

Our findings demonstrate the contributions of symbolic interactionist perspective to the analysis of migration-related decisions made by international students. We showed that the meaning students attach to the notion of “staying” is complex, which may be missed in cross-sectional surveys examining international students’ plans to stay in the host country or come back home upon graduation. While traditional migration literature explored the process of international movement as unidirectional (Baruch et al., 2007; Bratsberg, 1995; Lu et al., 2009), more recent scholarship highlights the complexity of the global movement of human capital and transnational mobilities (Sage et al., 2013; Szelényi, 2006). Our findings showed that the decision-making process employed by international students is tied to the experiences they acquire in Canada and is based on a variety of push and pull factors that work in complex tandem to shape students’ migration choices (Mazzarol & Soutar, 2002). For instance, students may be “pulled” by familial obligation to stay in Canada (to secure a better future for their children) and, at the same time, to go back home (to help aging parents). Similarly, the prospect of employment opportunities may attract students to stay in Canada, return back home, or move to another country. Consistent with findings from other studies, we demonstrated that international students’ migration-related plans may change with time (Hazen & Alberts, 2006). We also showed that students’ experiences in Canada may be instrumental in shaping their decision-making processes.

Our study has some limitations. Our participants came from 23 different countries with different social and cultural understandings about education, family obligations, or migration. Different laws regarding dual citizenship and ease of movement to and from the home country might have their own influences on students’ decision to stay in Canada. The diversity of our sample enabled us to identify some of the common factors shaping students’ decision-making process, but striving to summarize commonalities in the views of our participants, we did not delve into cultural differences that shaped students’ perceptions about their transition to Canada. We also did not explore how students’ level of study—undergraduate or graduate degree—shaped their decision-making processes about staying in Canada and how their duration of staying in Canada may have shaped their choices. Finally, we did not explore the role gender plays in shaping the decision to stay in Canada or going back home. Given the traditional gender roles assigned to familial responsibilities and the role culture plays in establishing these gender roles, it is possible that such nuanced analysis would have allowed us to explore the intersection between gender, culture, and migration, which is something that we aim to do in our future work.

he qualitative nature of our study mandates that the findings from this study would not be considered generalizable to the general population of international students in Canada. Rather, summarizing the results from our study, we aimed to highlight the subjective experiences of those students who took part in our study. While the conclusions drawn from our study cannot be considered representative of all international students in Canada, we believe that they, nonetheless, shed some light on how the decision-making process regarding migration can be experienced by some international students.

## Conclusion

In this study, we explored how international students make decisions about staying in Canada or going back home upon graduation and what factors shape their decision-making process. We showed that students assign various meanings to “staying” in Canada, ranging from remaining in Canada for a few additional months to moving to Canada permanently. We also showed that only a handful of students remained firm in their decision to stay or go back home with the vast majority altering their decision-making process based on their experiences of studying in Canada. Overall, our findings suggest that the considerable range of students’ views on staying in Canada may be a reflection of how education and immigration are understood by these students—they see themselves as a part of a globalized and mobile workforce where the boundaries between countries can bend with the right education, professional qualifications, and employment experiences. And it is possible that countries competing for international human capital might need to consider what temporary and permanent migration opportunities should be developed to attract “the best and the brightest” (Geddie, 2014) who are constantly on the move.
